# Ageing Increases Cardiac Electrical Remodelling in Rats and Mice via NOX4/ROS/CaMKII-Mediated Calcium Signalling

**DOI:** 10.1155/2022/8538296

**Published:** 2022-03-28

**Authors:** Xian Luo, Wendie Yu, Zhu Liu, Zhaoli Pu, Ting Liu, Yangpeng Li, Weichao Liu, Ming Lei, Xiaoqiu Tan, Tangting Chen

**Affiliations:** ^1^Key Laboratory of Medical Electrophysiology of Ministry of Education, Medical Electrophysiology Key Laboratory of Sichuan Province, Institute of Cardiovascular Research, Southwest Medical University, Luzhou, Sichuan 646000, China; ^2^Department of Cardiology, The Affiliated Hospital of Southwest Medical University, Luzhou, Sichuan 646000, China; ^3^Department of Pharmacology, University of Oxford, Mansfield Road, Oxford OX1 3QT, UK; ^4^Department of Physiology, School of Basic Medical Science, Southwest Medical University, Luzhou, Sichuan 646000, China

## Abstract

**Objective:**

Ageing is one of the risk factors associated with cardiovascular diseases including cardiac arrhythmias and heart failure. Ageing-related cardiac dysfunction involves a complicated pathophysiological progress. Abnormal membrane voltage and Ca^2+^ dynamics in aged cardiomyocytes contribute to ageing-related arrhythmias. However, its underlying mechanisms have not been well clarified.

**Methods:**

Young and old rats or mice were included in this study. Cardiac electrophysiological properties and functions were assessed by ECG, echocardiography, and ex vivo heart voltage and Ca^2+^ optical mapping. Proteomics, phosphor-proteomics, Western blotting, Masson staining, and ROS measurement were used to investigate the underlying mechanisms.

**Results:**

Ageing increased the incidence of cardiac hypertrophy and fibrosis in rats. Moreover, ageing increased the occurrence of ventricular tachycardia or ventricular fibrillation induced by rapid pacing and during isoprenaline (ISO) (1 mg/kg i.p.) challenge in mice *in vivo*. Optical mapping with dual dyes (membrane voltage (*V*_*m*_) dye and intracellular Ca^2+^ dye) simultaneously recording revealed that ageing increased the action potential duration (APD) and Ca^2+^ transient duration (CaTD) and slowed the ventricular conduction with the Langendorff-perfused mouse heart. More importantly, ageing increased the ISO-induced (1 *μ*M) changes of APD (*Δ*APD80) and CaTD (*Δ*CaTD50). Ageing also delayed the decay of Ca^2+^ transient by extending the decay time constant from 30% to 90% (*τ*_30−90_). In addition, ageing decreased the *V*_*m*_/*Ca*^2+^ latency which represented the coupling of *V*_*m*_/*Ca*^2+^ including between the midpoint of AP depolarization and Ca^2+^ upstroke, peak transmembrane voltage and peak cytosolic calcium, and time to 50% voltage repolarization and extrusion of cytosolic calcium. Optical mapping also revealed that ageing increased the ISO-induced arrhythmia incidence and occurrence of the excitation rotor. Proteomics and phosphor-proteomics assays from rat hearts demonstrated ageing-induced protein and phosphor-protein changes, suggesting that CaMKII was involved in ageing-induced change. Ageing increased the level of ROS and the expression of NOX4, oxidative CaMKII (ox-CaMKII), phosphorated CaMKII (p-CaMKII), and periostin.

**Conclusion:**

Ageing accelerates cardiac remodelling and increases the susceptibility to ventricular arrhythmias through NOX4/ROS/CaMKII pathway-mediated abnormal membrane voltage and intracellular Ca^2+^ handling and *V*_*m*_/*Ca*^2+^ coupling.

## 1. Introduction

Ageing has been demonstrated to be one of the major risk factors for cardiovascular disease (CVD) [1, 2], including the incidence of hypertension, arrhythmias, and heart failure, which contribute to higher morbidity and mortality in the elder population. Within the elderly people (>70 years), the proportion of total deaths worldwide due to CVD will reach up to 40% in 2030 from the World Health Organization (WHO) [3]. The heart has complicated pathophysiological changes during ageing. Considerable evidence implicates that endothelial dysfunction, mitochondrial oxidative stress, chromatin remodelling, and genomic instability are involved in vascular and cardiac dysfunction in elderly patients [4, 5].

Ageing is associated with the increased predisposition of arrhythmias through complicated structural and functional alterations in cardiac mechanical and electrical systems, as well as energetics and metabolism [1]. Myocardium hypertrophy and interstitial fibrosis—resulting in altered cellular coupling and exaggerated directional differences in conduction (anisotropy)—increase heterogeneity in impulse propagation properties and refractoriness of the myocardium [6, 7].

Mitochondria are the critical organelle for the generation of metabolic energy in eukaryotic cells through oxidative phosphorylation. More than 90% of the intracellular ATP consumed by cardiomyocytes was generated in mitochondria. The dysfunction of mitochondria has been demonstrated to be involved in multiple heart diseases [8–11]. The increase in oxidative stress due to the increase in reactive oxygen species (ROS) production with age results in an overall enhancement in the rate of cardiomyocyte death and increased fibrosis. Oxidative stress is believed to be an independent mediator of age-related arrhythmias through reducing the repolarization reserve and formation of early and delay afterdepolarizations due to activation of the Na^+^-Ca^2+^ exchanger. Lots of studies also revealed that Ca^2+^/calmodulin-dependent protein kinase II (CaMKII) activation contributed to a variety of cardiac diseases including heart failure and arrhythmias [12]. ROS and CaMKII played a crucial role in the development of arrhythmias. Xie et al. found that ROS-induced arrhythmias were blocked through inhibiting CaMKII [13]. It has been shown that the increased oxidative stress prolonged diastolic relaxation through oxidative damage of the sarcoplasmic reticulum SERCA ATP pump and decreased its Ca^2+^-sequestering activity [14]. The normal coupling and dynamics of membrane voltage (*V*_*m*_) and intracellular Ca^2+^ contributed to integrated cardiac electrophysiological function, whereas its abnormal coupling was involved in the occurrence of arrhythmias [15]. However, the molecular bases and underlying mechanisms for the increased susceptibility to arrhythmias in the elderly are not fully understood and need further exploration.

In this present study, we explored the role of oxidative stress in increased susceptibility to ventricular arrhythmias in ageing hearts and its underlying signalling pathway. We revealed the abnormal coupling of *V*_*m*_ and intracellular Ca^2+^ handing in ageing animals under ISO-induced *β* adrenergic stress conditions.

## 2. Methods and Materials

### 2.1. Animals

Young (4 months) and old (18 months) Wistar Kyoto (WKY) rats were purchased from Beijing Vital River Laboratory Animal Technology Co. Ltd. (Beijing, China). Young (2–3 months) and old (14–16 months) C57/BL mice were acquired from the Animal Centre of Southwest Medical University (Sichuan, China). Experimental protocols were performed with the approval of the Animal Care and Use Committee of the Southwest Medical University (no. 20160930).

### 2.2. Echocardiography and Hemodynamic Analysis in Intact Hearts

WKY rats were terminally anaesthetized with 2% isoflurane using a gas anaesthesia machine (RWD Life Science, Shenzhen city, Guangdong province, China). Parasternal short-axis section (PSAX) M-mode echocardiography was recorded using the Vevo®3100 micro-ultrasound imaging system (FUJIFILM VisualSonics Inc., Canada) following the manufacturer's instructions. Three measurements were taken at end-systole (s) and end-diastole (d) which were averaged to calculate the corresponding values of the intraventricular septal thickness (IVSs and IVSd), left ventricular posterior wall thickness (LVPWs and LVPWd), and left ventricular end-diastolic (LVIDd) and end-systolic dimensions (LVIDs). The ejection fraction (EF) and fractional shortening (FS) were also acquired from the recorded measurements.

### 2.3. Masson's Trichrome Staining

Hearts were removed from WKY rats anaesthetized with 2–5% isoflurane and transferred to 4% polyformaldehyde in PBS for fixation. 5 *μ*m paraffin sections were stained using Masson's trichrome method as described [16]. Images were acquired using PANNORAMIC SCAN (3DHISTECH Ltd., Hungary) to scan the whole film. The mean cross-sectional area of cardiomyocytes was calculated through approximately 400 randomly selected cells using CaseViewer software (version 2.3, 3DHISTECH Ltd., Hungary). The collagen volume fraction was measured through 20 images from different fields by ImageJ software (version 1.53e).

### 2.4. Electrocardiography (ECG) and Programmed Electrical Stimulation (PES)

Surface ECG recording of C57/BL mice *in vivo* was monitored using a multichannel physiological instrument (MP150, BIOPAC Systems Inc., USA) under anaesthesia with 0.5–1% isoflurane using the gas anaesthesia machine. To assess propensity to ventricular arrhythmias, anaesthetized mice were subjected to PES *in vivo*. Briefly, the heart was exposed and a stimulus electrode was placed on the surface of the heart and connected to the stimulator (SEN-7203, Nihon Kohden, Japan). Mice were subjected to burst pacing for 50 beats with cycle lengths (CLs) of 90, 70, 50, and 30 ms to induce the arrhythmia. Ventricular tachycardia with regular waveforms was defined as VT, while VF was characterized by irregular fibrillating waveforms. Intraperitoneal injection of 1 mg/kg isoproterenol (ISO) (cat: HY-B0448, MedChemExpress, USA) was performed to increase the susceptibility to arrhythmia.

### 2.5. Optical Mapping in Intact Hearts

The electrophysiological function and arrhythmia susceptibility of intact heart ex vivo were assessed using the optical mapping system equipped with an EMCCD camera according to our previous research [17, 18]. In brief, C57/BL mice were anaesthetized with 2–5% isoflurane followed by heparin injection (200 units intraperitoneal injection). The heart was removed and placed in cold PSS equilibrated with 95% O_2_ and 5% CO_2_. Using the Langendorff equipment, the aorta perfusion was performed at the 68–74 mmHg (1–2 ml/min) pressure at 37°C [18]. The Ca^2+^ dye Rhod-2AM (Thermo Fisher Scientific, USA) with 50 *μ*l bolus (stock solution: 1 mg/ml in dimethylsulfoxide) over a 5 min period and the voltage-sensitive dye RH237 (Thermo Fisher Scientific, USA) with a 30 *μ*l bolus of 1 mM concentration were loaded. After dye loading, the heart was moved to a special chamber in Krebs solution containing 10 *μ*M blebbistatin (Tocris Bioscience, Minneapolis, MN, USA), a myosin II inhibitor used to stop contractions and avoid movement artefacts. Then, the upright microscope equipped with a high-speed EMCCD (Evolve 512, Photometrics, Tucson, AZ, USA) camera was used for optical mapping. A four-light-emitting diode MacroLED lamp (530 nm, Cairn Research) offered the excitation light. *V*_*m*_ emission was collected using a 630 nm longpass filter while Ca^2+^ fluorescence was collected using a 585/40 nm bandpass filter. Data were acquired at 1000 frames/s in a 512 × 512 pixel grid. The heart was paced at ventricular apex with a CL of 100 ms (10 Hz) throughout the duration of the experiment. To assess the arrhythmia propensity, PES on the ventricular apex with CLs from 100 ms to 20 ms was subjected. Recorded image files are loaded into an optical mapping analysis software ElectroMap [19].

### 2.6. Proteomics and Phosphoproteomics Study

Hearts samples in WKY rats were acquired and treated with liquid nitrogen quickly and then kept at −80°C. Each group included three individual heart samples. About 150 mg of ventricle tissue for each sample was extracted for protein preparation that was used to multiomics array including proteomics and phosphoproteomics constructed in Novogene Co. Ltd. (Beijing, China). TMT labelling of peptides, separation of fractions, peptide/protein identification and quantification, transition library construction, and the functional analysis of protein and differentially expressed proteins (DEP) for proteomics were conducted according to the standard experiment and analysis protocol. In addition, phosphopeptide enrichment using phos-select iron affinity gel, motif analysis and kinase-substrate relationship analysis were carred out for phosphoproteomics study.

### 2.7. Measurement of Reactive Oxygen Species (ROS)

To measure ROS levels in C57/BL mouse heart tissue, 10 *μ*m slices were incubated with dihydroethidium (DHE. cat. no. HY-D0079, MedChemExpress, USA) for 30 min at 37°C and washed twice with PBS. Fluorescent signals at excitation/emission 535/610 nm were recorded using a fluorescence microscope (Olympus IX83, Japan). The average fluorescence intensity was calculated by ImageJ software.

### 2.8. Western Blotting

50 *μ*g total protein for each lane was separated using 5% stacking gel and 10% separation gel and transferred to a 0.45 *μ*m PVDF membrane (Millipore, USA). The membrane was incubated in TBST containing 5% nonfat milk for 2 h at room temperature to block nonspecific binding and was incubated with the primary antibody overnight at 4°C. The primary antibodies for NOX2 (cat. no. ab129068, Abcam, UK, 1 : 1000), NOX4 (cat. no. 14347-1-AP, ProteinTech, USA, 1 : 1000), oxidated CaMKII (ox-CaMKII) (cat. no. GTX36254, GeneTex, USA, 1 : 1000), phosphorylated CaMKII (p-CaMKII) (T287) (cat. no. ab182674, Abcam, UK, 1 : 1000), total CaMKII (t-CaMKII) (cat. no. ab22609, Abcam, UK, 1 : 1000), periostin (cat. no. ab92460, Abcam, UK, 1 : 1000), and the internal control antibody GAPDH (Santa Cruz Biotechnology, USA, 1 : 1000) were used. The membrane was incubated with the goat anti-mouse IgG HRP (BBI Life Sciences, China) and goat anti-rabbit IgG HRP (BBI Life Sciences, China) secondary antibody (1 : 1000) for 1 h at room temperature. The membrane was incubated in chemiluminescent HRP substrate (Millipore, USA) at room temperature for 5 min and then imaged with the Universal Hood II System (Bio-Rad, USA).

### 2.9. Statistical Analysis

Data from comparing the different experimental groups were presented as *means* ± *SEM* and analyzed using GraphPad Prism 8 software (GraphPad Software, San Diego, CA) by analysis of two-way variance (ANOVA), *t*-test, or chi-square test. A *P* value of <0.05 was considered statistically significant.

## 3. Results

### 3.1. Ageing Increased Cardiac Hypertrophy and Fibrosis

**Figure 1:**
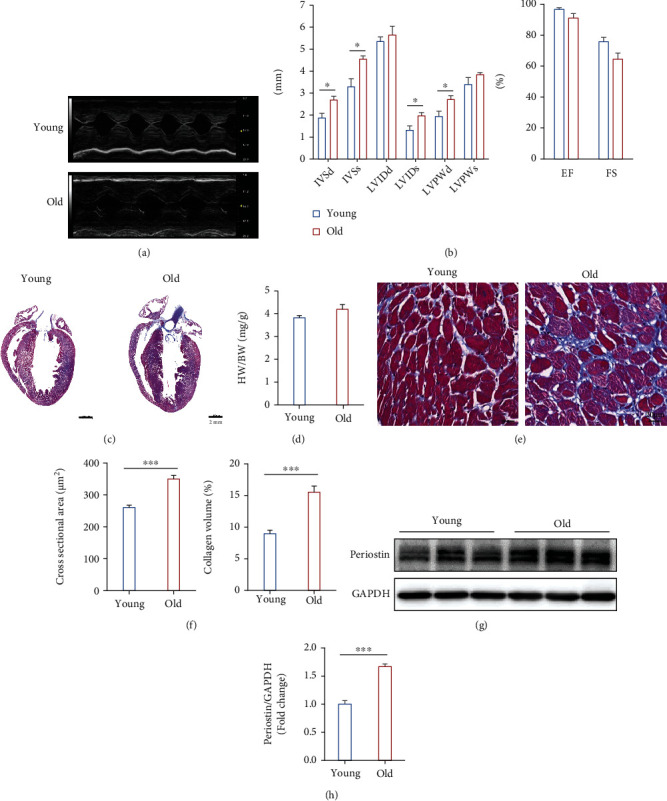
Effects of ageing on cardiac function, hypertrophy, and fibrosis. (a, b) The echocardiography showing the effect of ageing on cardiac function (*n* = 5). (c) Masson staining of cardiac coronal sections in young and old heart tissues, scale bar: 2 mm. (d) Histogram of heart weight/body weight ratios (HW/BW) (*n* = 5). (e) Masson staining of cardiac cross-sections, scale bar: 20 *μ*m. (f) Histograms of the cross-sectional area (*n* = 400, *N* = 3 each group) and collagen volume (*n* = 20, *N* = 3 each group). (g) Original image of Western blotting showing the band of Periostin (Postn) and GAPDH. (h) Histograms of the protein expression level of periostin (Postn). *n* = 6 (young group), 8 (old group). Compared with the young group, ^∗^*P* < 0.05, ^∗∗^*P* < 0.01, and ^∗∗∗^*P* < 0.001.

We first explored the cardiac functions and basic characters of hearts from young and old WKY rats by echocardiography and Masson staining as shown in Figure 1. Echocardiography and hemodynamic analysis showed that IVSd, IVSs, LVIDd, LVIDs, LVPWd, and LVPWs were increased in old rats (Figures 1(a) and 1(b)). IVSd, IVSs, LVIDs, and LVPWd have a statistical difference between the two groups (*P* = 0.017, *P* = 0.015, *P* = 0.031, and *P* = 0.032, respectively), while EF and FS in old rats were slightly decreased with no statistical difference (*P* = 0.103, *P* = 0.051). In addition, we found heart enlargement and collagen deposition in histology. Because the older mice gained weight, there was no significant increase in the HW/BW ratio between young and old rats (*P* = 0.070). However, the cross-sectional area and collagen volume were increased in the old group (both *P* ≤ 0.001). Furthermore, the expression of periostin, which is one of the markers of cardiac fibrosis, also significantly increased in the old group (*P* ≤ 0.001, Figures 1(g) and 1(h)). These data suggest that ageing exacerbated cardiac hypertrophy and fibrosis. These data suggest that ageing exacerbated cardiac hypertrophy and fibrosis.

### 3.2. Ageing Increased the Susceptibility to Ventricular Arrhythmias Induced by Rapid Pacing *In Vivo*

**Figure 2:**
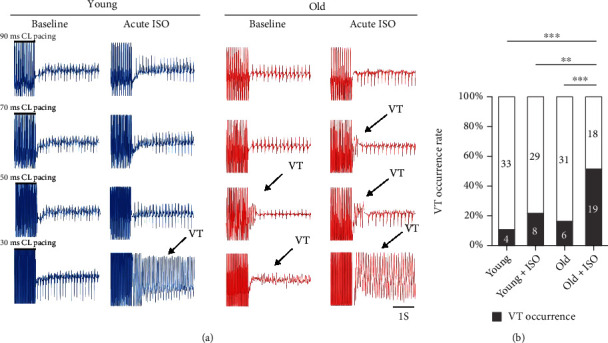
ISO increased cardiac arrhythmias incidence in old mice *in vivo*. (a) The typical ECG recording showing that ageing increased susceptibility to ISO-induced arrhythmias using PES with PCL from 90 ms to 30 ms. (b) Histogram of the VT occurrence rate in young and old mice with or without treatment of ISO (1 mg/kg, i.p.) (^∗∗^*P* < 0.01 and ^∗∗∗^*P* < 0.001).

Previous studies have demonstrated that cardiac fibrosis exists in various cardiovascular diseases and accelerates the occurrence and maintenance of cardiac arrhythmias [15, 20]. Therefore, we further investigated the effects of ageing on the susceptibility to ventricular arrhythmias using surface ECG and PES with a cycle length from 90 ms to 30 ms to induce VT in C57/BL mice *in vivo*. At baseline, there was no significant difference in ECG basic characteristics between the young and old groups (Figure 2). There was also no significant difference in the incidence of arrhythmia with PES between the young (10.81%, *n* = 4/37) and old (16.22%, *n* = 6/37) groups in the baseline (*P* = 0.496). However, after acute intraperitoneal injection of ISO (1 mg/kg) for 5 min, the incidence of VT in the old group (51.35%, *n* = 19/37) was significantly increased than that in the young group (21.62%, *n* = 8/37) (*P* = 0.008). The data suggest that the old mice are more susceptible to cardiac arrhythmia under the acute ISO-induced *β* adrenergic stress condition.

### 3.3. Ageing Induced Abnormal Action Potential (AP) and Ca^2+^ Signal

**Figure 3:**
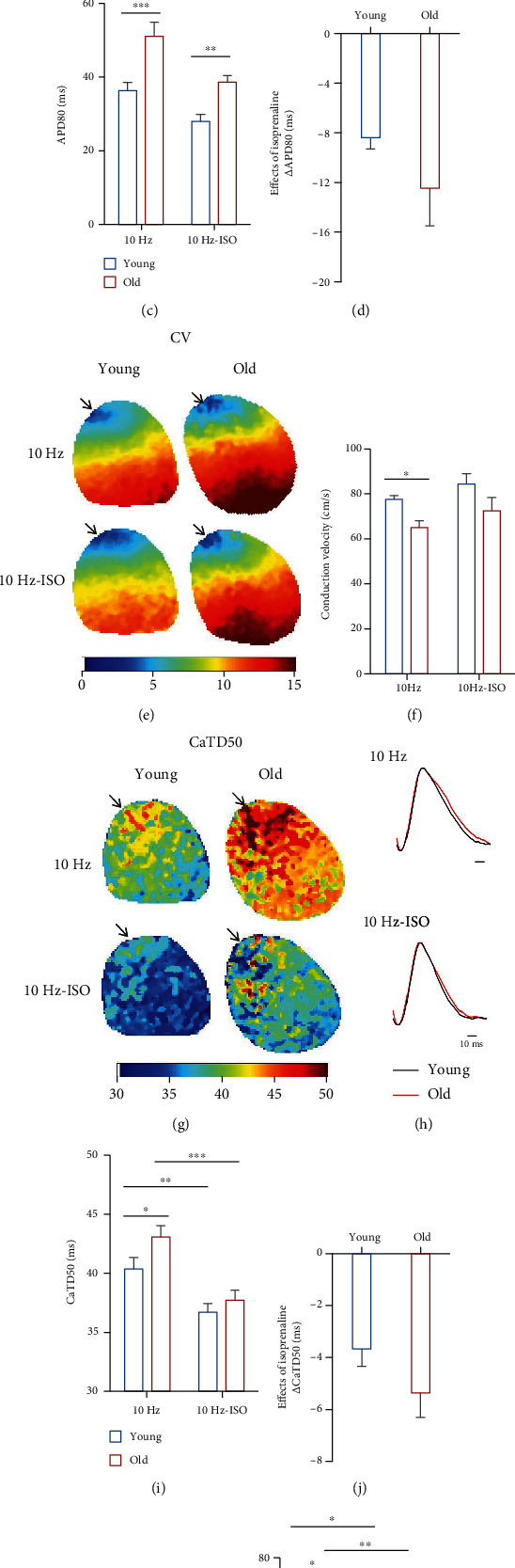
Optical mapping showed that ageing modulated AP and calcium duration. (a, b) The typical APD_80_ map and AP trace (young: black line; old: red line) in intact Langendorff perfused young and old mouse hearts before and following 1 *μ*M ISO challenge in pacing at 10 Hz. (c) Quantitative analysis of APD_80_ from individual hearts in young and old mice before and following 1 *μ*M ISO (^∗^*P* < 0.05, ^∗∗^*P* < 0.01, and ^∗∗∗^*P* < 0.001). (d) Ageing induced fractional increases in APD_80_ challenged by ISO (*Δ*APD_80_) (*P* > 0.05). (e) The typical CV map. (f) Quantitative analysis of APD_80_ from individual hearts in young and old mice before and following 1 *μ*M ISO (^∗^*P* < 0.05). (g, h) The typical CaTD_50_ map and calcium transient trace (young: black line; old: red line) in intact Langendorff perfused young and old mouse hearts before and following 1 *μ*M ISO challenge in pacing at 10 Hz. (i) Quantitative analysis of CaTD_50_ from individual hearts in young and old mice before and following 1 *μ*M ISO ^∗^*P* < 0.05, ^∗∗^*P* < 0.01, and ^∗∗∗^*P* < 0.001). (j) Ageing induced fractional changes in APD_80_ challenged by ISO (*Δ*CaTD_50_) (*P* > 0.05). (k) The representative CaTD map shows the calculation of decay constant (*τ*) by fitting of exponential decay points between 30% and 90% decay from the peak (*τ*_30__–90_), illustrated for the regional signal in red. (l) Histogram showing the *τ*_30__–90_ in young and old mice before and following 1 *μ*M ISO pacing at 10 Hz (^∗^*P* < 0.05 and ^∗∗^*P* < 0.01). *n* = 5 (each group).

**Figure 4:**
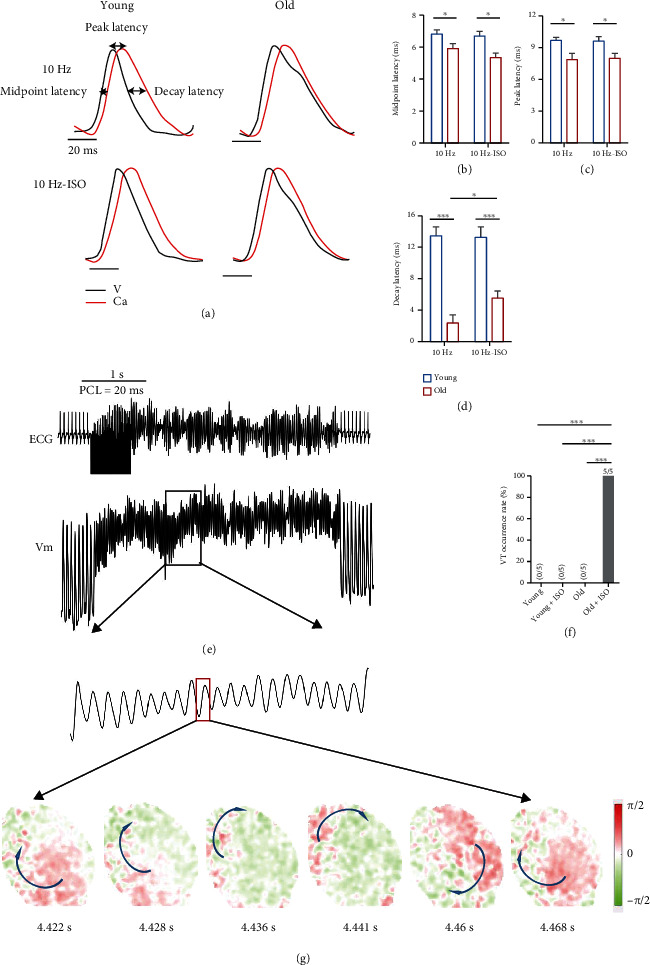
Optical mapping showed *V*_*m*_/Ca^2+^ latency and cardiac arrhythmias: (a) The representative map shows calculation of *V*_*m*_/Ca^2+^ latency (at upstroke, peak, and decay time points). (b) Statistical data of midpoint latency. (c) Statistical data of peak latency. (d) Statistical data of decay latency. (e) ECG and Vm of arrhythmia. Isochrone maps corresponding to the period marked by a red square in (e) (mapping trace). Arrows indicate the directions of wavefront propagation. (f) Histogram of the VT occurrence rate in young and old mice with or without treatment of ISO. (g) The phase heat map showed the rotor of arrhythmias (^∗^*P* < 0.05, ^∗∗^*P* < 0.01, and ^∗∗∗^*P* < 0.001), *n* = 5 (each group).

Cardiac fibrosis can cause abnormal electrical activity and conduction, which contributed to cardiac arrhythmia [6, 21, 22]. Therefore, we explored optical mapping to investigate the effects of ageing on AP and the Ca^2+^ signal and their coupling with programmed electrical stimulation (PES) with PCL of 100 ms (pacing frequency: 10 Hz) at the cardiac apex as shown in Figures 3 and 4. The APD80 (the duration from the peak of AP to repolarization at 80%) (Figure 3(a)) and CaTD50 (the duration from the time of the maximum upstroke velocity to decay at 50% of Ca^2+^ transient) (Figure 3(g)) were quantified as AP duration and Ca^2+^ transient duration, respectively. We found that the APD80 in old mice was significantly prolonged from 36.31 ± 2.28 *ms* to 51.10 ± 3.81 *ms* (*P* ≤ 0.001) pacing at 10 Hz (Figure 3(c)). After ISO perfusion, the APD80 was shortened in both young and old mice (27.94 ± 1.94 *ms* in the young group, 38.63 ± 1.78 *ms* in the old group, *P* = 0.010) and it is interesting that the ISO-induced decrease of APD80 (quantified as *Δ*APD80) was more obvious in the old mice with *Δ*APD80 from −8.38 ± 0.93 ms to −12.46 ± 3.03 ms (Figure 3(d)).

We further analyzed the calculation of “conduction velocity” (CV) to provide a measure of AP activation spread and conduction across the whole tissue according to the previous protocol [19] as shown in Figure 3(e). We found that CV was decreased from 77.46 ± 1.71 *cm*/*s* in young mice to 64.93 ± 3.12 *cm*/*s* in old mice pacing at 10 Hz (*P* = 0.042), Figures 3(e) and 3(f)). These data suggest that ageing inhibits the AP spread and conduction.

We also observed the Ca^2+^ transient duration and found that CaTD50 in the old mice were also prolonged compared to the young mice from 40.37 ± 0.97 *ms* to 43.09 ± 0.94 *ms* (*P* = 0.044) pacing at 10 Hz (Figures 3(g)–3(i)). Similar to the effect of APD80, CaTD50 was significantly shortened after ISO perfusion (36.70 ± 0.74 *ms* in the young group, 37.72 ± 0.85 *ms* in the old group) (Figure 3(i)). The ISO-induced decrement of CaTD50 (quantified as *Δ*CaTD50) was more obvious in the old mice from −3.67 ± 0.68 *ms* to −5.36 ± 0.94 *ms* (Figure 3(j)). We further fitted the decay curve of the Ca^2+^ transient and calculated the time constant (Tau) from decay at 30% to at 90% (Tau30-90, *τ*_30__−90_) (Figure 3(k)), which represented the late diastolic phage of calcium transient. The results showed that *τ*_30__−90_ was increased in old mice pacing at 10 Hz (55.78 ± 4.06 in the young group, 67.93 ± 2.68 in the old group, *P* = 0.014) (Figure 3(l)). Treatment of ISO also accelerated Ca^2+^ decay both in young and old mice (43.38 ± 1.98 in the young group, 54.78 ± 3.36 in the old group, *P* = 0.020) (Figure 3(l)).

Abnormal coupling of membrane voltage (*V*_*m*_) and intracellular Ca^2+^ transient dynamics is the important mechanism of cardiac arrhythmias [15]. To explore Ca^2+^ and *V*_*m*_ clock coupling and how ageing may affect such coupling, we analyzed the voltage-calcium latency as optical mapping was simultaneously recorded in *V*_*m*_ and Ca^2+^ at upstroke, peak, and decay time points as shown in Figures 4(a)–4(d). Old mice demonstrated a significantly shortened latency time between the midpoint of AP depolarization and Ca^2+^ upstroke (6.81 ± 0.27 *ms* in the young group, 5.91 ± 0.29 *ms* in the old group, *P* = 0.029), peak transmembrane voltage and peak cytosolic calcium (9.67 ± 0.30 *ms* in young group, 7.87 ± 1.31 *ms* in the old group, *P* = 0.010), and time to 50% voltage repolarization and extrusion of cytosolic calcium (13.44 ± 1.15 *ms* in the young group, 2.33 ± 0.63 *ms* in the old group, *P* ≤ 0.001).

In addition, we explored the arrhythmia incidence with optical mapping following 1 *μ*M ISO treatment pacing at 50 Hz ex vivo. Old mice presented a high incidence of VT events (Figure 4(e)). Under baseline without treatment of ISO, both young and old mice did not have arrhythmias. However, 1 *μ*M ISO induced 100% (5/5) VT occurrence in old mice, while there was no arrhythmia in young mice treated with ISO (Figure 4(f)). These data were consistent with the surface ECG recording *in vivo* (Figure 2). The heat map showed the detail of the rotor and the process of VT (Figure 4(g)).

All the data with optical mapping suggest that ageing increases the arrhythmia incidence through increasing the AP and Ca^2+^ duration and shortening the latency.

### 3.4. The Effects of Ageing on Proteomics and Phosphoproteomics

**Figure 5:**
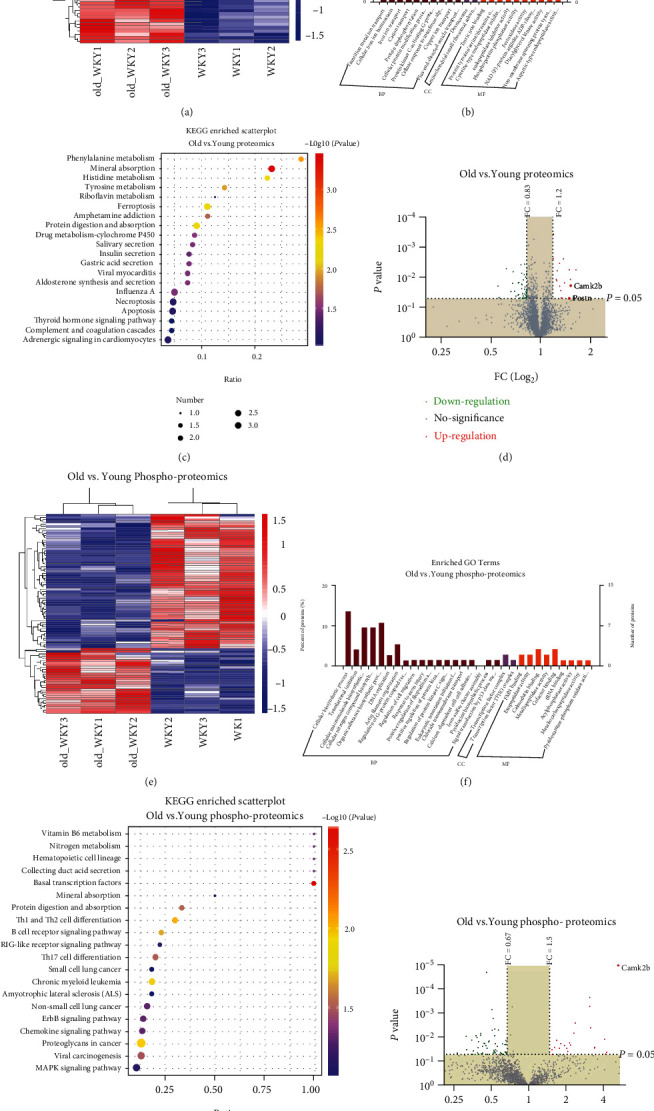
Proteomics and phosphoproteomics arrays revealed that ageing induced the changes of protein and phosphoprotein. (a) Heatmap of differential protein in young and old rat hearts. (b, c) GO and KEGG enrichment of differential protein in young and old rat hearts. (d) Volcanic map of differential phosphoprotein in young and old rat hearts showing that CaMKII and Postn were upregulated in heart tissue from old rats. (e) Heat map of differential phosphoprotein in young and old rat hearts. (f, g) GO and KEGG enrichment of differential phosphoprotein in young and old rat hearts. (h) Volcanic map of differential phosphoprotein in young and old rat hearts showing that phospho-CaMKII was upregulated in heart tissue from old rats. *n* = 3 (each group).

To further identify the specific mechanism of age-induced fibrosis leading to arrhythmias, we used proteomics and phosphoproteomics to analyze changes in protein expression in both groups. By using the LC-MS/MS analysis combined with TMT labelling technology, we identified a total of 3896 proteins that were common to the young and old WKY rat hearts. Proteins with *P* < 0.05 and *FC* ≥ 1.2 or *FC* ≤ 0.83 were considered differentially expressed. In addition, using phos-select iron affinity gel and motif analysis, we identified a total of 1475 phosphoproteins which were common to the young and old WKY rat hearts. Phosphoproteins with *P* < 0.05 and *FC* ≥ 1.5 or *FC* ≤ 0.67 were considered differentially expressed. As shown in Figure 5, ageing induced significant changes in proteins and phosphoproteins. As shown in the heat map in Figures 5(a) and 5(e), compared with young rats, the expression profile in old rats induced 58 proteins and 89 phosphoproteins in proteomics and phosphoproteomics array. Gene ontology analysis (Figure 5(b)) in proteomics was performed to reveal the strongly enriched biological processes: ion translation process, cellular protein modification process, and protein kinase C-activating G-protein coupled receptor signalling pathway. In addition, we also revealed the strongly enriched molecular function of differential proteins: protein tyrosine/serine/threonine activity, phosphoprotein phosphatase activity, ferroxidase activity, and NAD(P) + protein-arginine ADP-ribosyl transferase activity. Further KEGG analysis (Figure 5(c)) indicates that these differential proteins are also highly enriched in some pathways associated with ageing: adrenergic signalling in cardiomyocytes, apoptosis, necroptosis, protein digestion and absorption, aldosterone synthesis and secretion, insulin secretion, ferroptosis, and viral myocarditis. Gene ontology analysis (Figure 5(f)) in phosphoproteomics was performed to reveal the strongly enriched biological processes: cellular biosynthetic process, translational initiation, cellular macromolecule biosynthetic process, cellular nitrogen compound biosynthetic process, organic substance biosynthetic process, DNA replication, actin filament organization, regulation of G-protein coupled receptor protein signalling pathway, regulation of cell migration, calcium-dependent cell-cell adhesion via plasma membrane cell adhesion molecules, and signal transduction by the p53 class mediator. Further KEGG analysis (Figure 5(g)) indicates that these differential proteins are also highly enriched in some pathways associated with ageing: proteoglycans, MAPK signalling pathway, viral carcinogenesis, chemokine signalling pathway, and ErbB signalling pathway. Comparing proteomics and phosphoproteomics data, we found that CaMKII expression increased in both proteomics and phosphoproteomics (Figures 5(d) and 5(h)).

### 3.5. The NOX/ROS/CaMKII Pathway Was Involved in the Effect of Ageing

**Figure 6:**
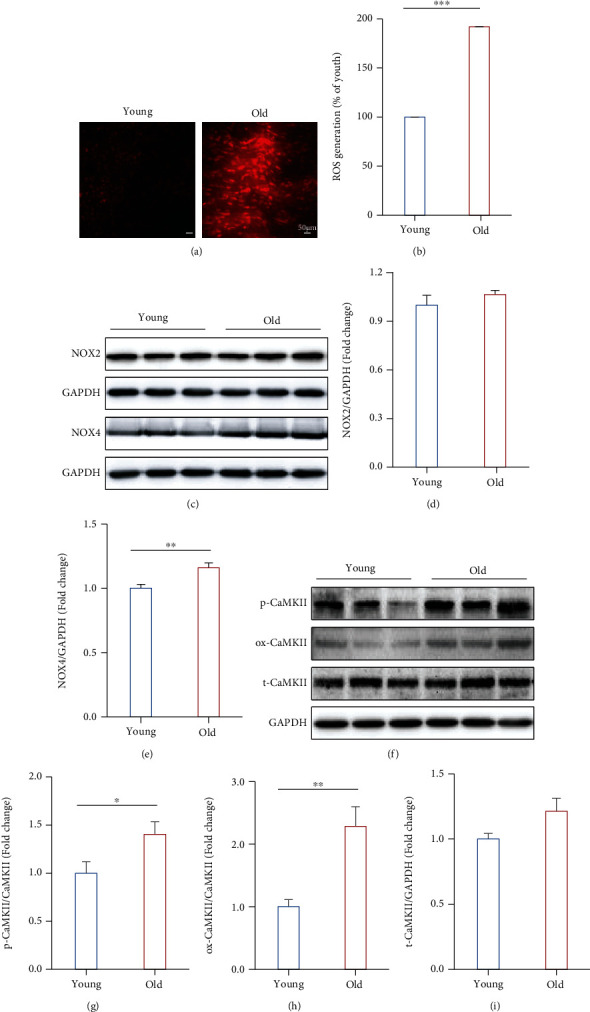
NOX4/ROS/CaMKII pathway was involved in the effect of ageing. (a) Typical image of DHE staining showing the ROS level in young and old mouse heart tissues. (b) Histogram of ROS generation in young and old mice (*P* < 0.001). (c, d) Original image of Western blotting showing the band of NOX4, NOX2 (c), p-CaMKII, ox-CaMKII, and t-CaMKII (f). (d–i) Histograms of the protein expression levels of NOX2 (*P* > 0.05), NOX4 (*P* < 0.01), p-CaMKII (*P* < 0.01), ox-CaMKII (*P* < 0.05), and t-CaMKII (*P* > 0.05). *n* = 6 (young group) and 8 (old group). Compared with the young group, ^∗^*P* < 0.05, ^∗∗^*P* < 0.01, and ^∗∗∗^*P* < 0.001.

To further confirm the proteomics and phosphoproteomics results which showed significant changes in the revealed abnormal expression of oxidative stress and CaMKII pathway between the young and old mice, the ROS was firstly determined by DHE staining and the results showed that intracellular fluorescence intensity of ethidium is significantly higher in the heart from old mice (*P* ≤ 0.001), indicating increased oxidative stress induced by ageing (Figures 6(a) and 6(b)). NOXs were the major source of ROS in cardiomyocytes. The expression of oxidative stress-related proteins NOX2 and NOX4 was detected by Western blot. The results demonstrated that ageing significantly increased the expression of NOX4 (*P* = 0.004) rather than NOX2 (*P* = 0.161) in the old group (Figures 6(c)–6(e)). Moreover, proteomics and phosphoproteomics data have demonstrated a significant increase in CaMKII expression and phosphorylation. So, the expression of p-CaMKII, ox-CaMKII, and t-CaMKII were detected by Western blot (Figures 6(f)–6(h)). The expression of p-CaMKII (*P* = 0.043) and ox-CaMKII (*P* = 0.003) was significantly increased in the old group, while there was no significant increase in the expression of t-CaMKII (*P* = 0.096) (Figure 6(i)).

## 4. Discussion

Ageing is one of the important risk factors for CVD and is associated with an increased prevalence of ventricular tachyarrhythmia, which contributed to higher morbidity and mortality in the elderly [1]. The prevalence and direct medical costs for CVD were significantly increased in over 65 years of age (especially over 80 years of age) in the United States [2]. Therefore, it is important to explore the mechanisms underlying the ageing process to find approaches to improve it. In this study, we revealed that cardiac remodelling and mitochondrial oxidative stress may contribute to ventricular tachyarrhythmia in the elderly by inducing abnormal Ca^2+^ and *V*_*m*_ coupling via the NOX4/ROS/CaMKII pathway.

It is well known that ageing induces cardiac remodelling even in the absence of underlying pathologies [23, 24]. Consistent with published results, we found that ageing exacerbates cardiac fibrosis and impaired cardiac functions in rats. Moreover, our ECG and PES experiments showed significantly increased susceptibility of aged hearts to rapid pacing-induced ventricular arrhythmias, which were in agreement with previous reports [25, 26]. In ventricular arrhythmias, fibrosis has been demonstrated to play important roles in creating an essential substrate for these arrhythmias persisting [27]. The published and our present data indicate that ageing-induced fibrosis may be involved in ventricular tachyarrhythmia.

The impairment of electrical activity and conduction has been identified to contribute to cardiac arrhythmia [6, 21, 22]. Using optical mapping assay, we found that the elderly hearts exhibited increased AP and Ca^2+^ duration and shortened *V*_*m*_/*Ca*^2+^ latency. Although there have been few studies to investigate the effects of ageing on *V*_*m*_/*Ca*^2+^ latency, growing evidence has identified abnormal myocardial AP and Ca^2+^ signal induced by ageing [26, 28], which are partly consistent with our results. Previous reports have shown that ATP-sensitive K^+^ channels, delayed rectifier K^+^ channels, and other potassium channels were dysfunctional in the ageing heart, which may contribute to the prolongation of APD and increased the susceptibility to arrhythmias [25, 29]. Furthermore, Lei and Huang recently suggested that normal cardiac excitation requires the cyclic events in the intracellular Ca^2+^ (Ca^2+^ clock) and membrane voltage (membrane clock) homeostasis to be aligned and disruption in this alignment leads to arrhythmia [15]. Our results and the published reports indicate that the shortened *V*_*m*_/*Ca*^2+^ latency and abnormal coupling of Ca^2+^ and *V*_*m*_ may be involved in the increased ventricular arrhythmias in ageing.

Mitochondria play important roles in cell processes and age-related pathological alterations of the heart. Ageing-related dysfunction of mitochondria contributed to the development of all common age-related diseases including fibrosis and arrhythmias. We found that the ROS level was increased in the elderly heart, which was also consistent with the previous study [30]. NADPH oxidases (NOXs) were the important source of ROS in the heart, which included NOX1 to 6 subtypes, and NOX2 and NOX4 were the important NOXs in the heart [31]. NOX2 was more expressed in the cell membrane, while NOX4 was predominantly expressed in mitochondria [31]. In our study, it is interesting that NOX4, not NOX2, was increased in the elderly heart (Figures 6(c)–6(e)), which suggested that NOX4 was the main source of increased ROS in the ageing heart. Moreover, it has been shown that ageing significantly inhibited the activity of enzymes that can scavenge ROS, such as SOD [32], indicating ageing-induced ROS generation may also be relative to SOD.

CaMKII, which plays a crucial role in cardiac normal function, can also be oxidized and activated by increased levels of ROS in various cardiac diseases [12, 33, 34]. There are lots of researches focusing on the activation of CaMKII through phosphorylation. In this study, our phosphoproteomics arrays and Western blotting experiments showed that not only the phosphorylated CaMKII but also the oxidative CaMKII were significantly increased in the elderly hearts, indicating that increased NOX4/ROS may also contribute to the activation of CaMKII through oxidation. Furthermore, CaMKII activation accelerated the Ca^2+^ release through phosphorylation of RYR2, which prolonged diastolic relaxation and may induce arrhythmias through DAD via activation of the Na + −Ca^2+^ exchanger. Taken together, NOX4/ROS-activated CaMKII may contribute to increased ventricular arrhythmias by inducing abnormal Ca^2+^ handling in elderly hearts. In addition, previous studies suggested that the activation of CaMKII was involved in cardiac fibrosis [35, 36]. Therefore, activated CaMKII may contribute to ageing-induced cardiac fibrosis and structural remodelling.

**Figure 7:**
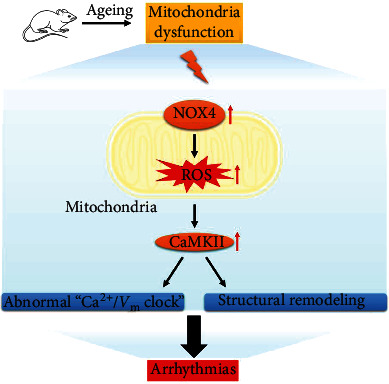
Schematic image of the signalling pathways of ageing-induced arrhythmias. Ageing increases the susceptibility to ventricular arrhythmias through NOX4/ROS/CaMKII pathway-mediated cardiac remodelling and abnormal *V*_*m*_/Ca^2+^ coupling.

NTotally, as shown in Figure 7, in this study, we revealed that cardiac remodelling and activation of the NOX4/ROS/CaMKII pathway contributed to ventricular arrhythmias in the elderly. More importantly, abnormal Ca^2+^ and *V*_*m*_ coupling were the important mechanisms involved in the susceptibility to ventricular arrhythmias in ageing.

## Data Availability

The data used to support the findings of this study may be released upon application to the corresponding author, Tangting Chen, who can be contacted through the following email: ctt@swmu.edu.cn
